# Determining the association of medical co-morbidity with subjective and objective cognitive performance in an inner city memory disorders clinic: a retrospective chart review

**DOI:** 10.1186/1471-2318-10-89

**Published:** 2010-12-17

**Authors:** Corinne E Fischer, Depeng Jiang, Tom A Schweizer

**Affiliations:** 1 Department of Psychiatry, room 17044, St. Michael's Hospital, #30 Bond St., Toronto, Ontario, M5B 1W8, Canada; 2 Department of Community Health Services, University of Manitoba Department of Community Health Services, Faculty of Medicine, University of Manitoba, Winnipeg, Manitoba, R3E 0W3, Canada; 3 Department of Neurosurgery, St. Michael's Hospital, #30 Bond St. Toronto, Ontario M5B 1W8, Canada; 4 Keenan Research Centre of the Li Ka Shing Knowledge Institute, room 2-30, #209 Victoria St., Toronto, Ontario, M5B 1T8, Canada

## Abstract

**Background:**

Medical co-morbidity may be associated with impaired cognitive function based on prior studies. However, no studies to date have determined to what extent this association is linked to medical illness or other factors that may be linked to medical illness (such as education, income levels, depression or subjective memory loss). The present study examined how medical co-morbidity, socioeconomic status (defined as residential SES), education and depression are associated with subjective and objective memory function in a sample of patients recruited from a university affiliated Memory Disorders Clinic located in a large Canadian inner city teaching hospital.

**Methods:**

Data was collected from 85 consecutive referrals to an Inner City Memory Disorders Clinic including socio-demographic characteristics, cognitive status and medical co-morbidity. Descriptive and correlational analyses were conducted.

**Results:**

Impaired objective cognitive function correlated significantly with increased medical co-morbidity and partially with education but not with residential SES or depression. Elevated memory complaints correlated significantly with depression, inversely with residential SES and not at all with medical co-morbidity or education.

**Conclusions:**

Increased medical co-morbidity is significantly associated with impaired cognitive performance but not with subjective memory complaints in an Inner City Memory Clinic sample.

## Background

Increased medical co-morbidity is commonly associated with the aging process [1,2]. There are many reasons for this. For example, age is a risk factor for many common diseases such as diabetes and chronic obstructive lung disease. According to previous research, medical co-morbidity rates tend to be higher among patients living in large urban centres, often due to low socioeconomic status and poorer access to health services [3]. High rates of medical co-morbidity have also been found to be associated with a number of adverse outcomes, including impaired function in activities of daily living, increased utilization of acute care services, and increased mortality [2,4,5]. The question of the impact of medical co-morbidity on cognitive function is a complex one. It is generally known that having multiple medical disorders may be associated with impaired brain function, partly due to the direct neurotoxic effects of disease on the brain and partly due to the possible neurotoxic effects of medications used to treat these diseases [6]. Patients with low SES may be particularly at risk [7,8]. In spite of this common assertion, there have been few studies that have systematically evaluated the impact of medical co-morbidity on brain function and no studies that have controlled for SES.

Of the existing studies that have examined the effect of medical co-morbidity on cognitive function, the majority have focused on objective function as opposed to subjective function. Lyketsos et al (2005) [9] examined a community sample and discovered medical co-morbidity was much higher among individuals with dementia. Cullum et al [10] examined a sample of older medical inpatients and discovered a strong correlation between medical co-morbidity and depression but surprisingly no significant correlation with cognition. It is possible that the lack of an association found in this study may be attributable to the low sensitivity of the cognitive instrument used (AMTS) in picking up changes in cognitive functioning. Mariani et al [11] examined a sample of patients with amnestic MCI (aMCI) and discovered cognitive functioning was a strong predictor of IADL performance while co-morbidity was not, possibly due to the fact that traditionally changes in ADL performance are more strongly linked to medical co-morbidity. Salvi et al [12] examined a cohort of acutely ill medical inpatients and found a strong correlation between medical co-morbidity, cognitive functioning, length of stay, depression and disability. Many studies have looked at the link between medical co-morbidity and depression in older adults, demonstrating a strong association [13-15]. However, no studies to date have explored how SES might impact on this association.

Subjective memory loss may or may not correlate with objective memory function based on previous research [16,17]. What is clear is that depressive symptoms and personality traits may mediate part of this discrepancy. In other words, patients with depressive symptoms may have elevated subjective memory complaints in the face of normal cognition [18,19]. The opposite pattern (normal subjective memory complaints in the face of objective memory function) may be observed in patients with poor frontal lobe function, such as patients with vascular dementia or advanced dementia [20,21]. Patients with elevated medical co-morbidity are at risk on both counts. First, medical co-morbidity has been found to be associated with high levels of depression [13-15]. Second, medical co-morbidity is often associated with dementia and cognitive dysfunction [9].

The objective of this study was to examine the impact of medical co-morbidity and other SES factors on subjective and objective memory function in a sample of patients with mixed diagnoses (dementia, MCI, normal cognitive functioning) referred to a Memory Disorders Clinic. As a result of it's geographic location the clinic sees patients with both high and low SES, making it an ideal environment to study the effects of SES. Furthermore, patients are referred to the clinic by their family doctor and/or specialist often with a complaint of memory loss but not necessarily objective findings. Thus, the base rates of memory complaints tends to be high and it provides an ideal opportunity for studying how SES and associated factors may modify the relationship between subjective and objective cognitive function in a memory clinic setting. We hypothesized based on the literature that patients referred to our Memory Clinic with low SES, increased medical co-morbidity, low education, and the presence of depression would be more likely to have objective cognitive impairment but not necessarily subjective memory loss.

## Methods

Data from 85 patients aged 50 or above attending a Memory Disorders Clinic located in a large urban centre was examined retrospectively. The sample consisted of community dwelling patients with a variety of diagnoses (dementia, MCI, cognitively normal) referred by their family physicians with a complaint of memory loss. While patients were all medically stable many were on a number of medications. The study was approved by the St. Michaels Hospital Research Ethics Board.

## Measures

### Residential SES

We chose to define SES based on neighbourhood income quintiles. Prior studies that have used residential SES as opposed to personal SES have typically been done on a much larger scale and have been used to determine the effect of neighbourhood SES on the prevalence of dementia, etc. We elected to use residential SES as opposed to personal SES (ie income levels) in order to maximize our clinical sample. Neighbourhood income quintiles were computed using existing census data and divided the region of Toronto into five quintiles. The quintiles ranged from one (lowest income) to five (highest income) and for the purposes of analysis we pooled the two lowest income groups and the three highest income groups with an income less than $35,000.00 being defined as the cut off for low versus high. For the purposes of our study we have decided to separate out education from SES.

### Cumulative Illness Rating Scale (CIRS)[22]

Medical co-morbidity was measured using the cumulative illness rating scale, geriatric version (CIRS-G). In this scale the body is divided into 13 different systems corresponding to 13 different organ areas. Based on the nature of the illness a severity score is assigned ranging from 0 (no impairment) to 4 (severe impairment). Certain illnesses that affect multiple organ systems may in some circumstances be coded more than once. The total score is then achieved by adding up the sub-scores for each organ system and indicates overall medical burden.

### Patient Assessment of Own Function (PAOF)[23]

Subjective memory complaints were measured using the Patient Assessment of Own Function (PAOF) [23]. The Patient Assessment of Own Functioning (PAOF) is a 33 item tool designed to measure subjective memory complaints. It includes questions about a variety of cognitive functions including memory, language and communication, sensory motor skills and higher level intellectual functions. Participants are asked to respond while thinking about the last two months and to rate their complaints on the 6 points scale. While it has not been validated in older patients with dementia it has been used in a number of cognitively impaired populations including those with psychiatric problems, HIV patients and those with chronic medical illnesses.

### Behavioural Neurology Assessment [24]

The BNA is a clinician administered cognitive test that measures multiple cognitive domains including language, attention, visual spatial function, naming and executive function. It is scored out of 114, with the cut off for dementia being low 80. It has been validated in patients with dementia and has been demonstrated to be superior to the MMSE in detecting dementia [24].

### Folstein Mini-Mental Status Examination [25]

The MMSE is a measure of global memory function [25]. It is scored out of 30 and measures a variety of cognitive functions including recall, orientation, visual spatial function, etc. It has been extensively used in dementia trials, with the cut off for dementia being 26 or less.

### Diagnosis of Dementia/Depression

A diagnosis of dementia was made following comprehensive assessment by a behavioural neurologist and geriatric psychiatrist in accordance with DSM-IV criteria. All patients had brain imaging and routine blood work to rule out reversible causes of dementia as well as cognitive testing using the BNA and MMSE. While cut off scores were not routinely used, most patients with dementia scored below 26 out of 30 on the MMSE or below 80 on the BNA. Depression was diagnosed using DSM-IV criteria following comprehensive assessment by a geriatric psychiatrist.

### Statistical methods

Continuous variables were summarized in the descriptive analyses using the mean, standard deviation and range. Categorical variables and ordinal variables were summarized using the number (percent) in various categories. Spearman rank correlations were used to examine the impact of education, socioeconomic status, depression and medical co-morbidity on subjective and objective memory function.

To avoid problems with confounders we ran two additional analyses. The first was a repeat of the spearman rank correlation analysis adjusting for the effects of age. The second was a multivariable linear regression analysis to determine if the effects remained when medical co-morbidity was removed from the analyses. Statistical analyses were performed using SAS Version 9.2 (SAS Institute, Cary, NC). All p-values were two-tailed and a p-value less than 0.05 was regarded as being significant.

## Results

In terms of descriptive statistics (see Table 1), the mean age of the sample was 69.2(10.0) years, the mean level of education was 14.0(3.5) years and the mean CIRS score was 5.2(2.9), suggesting most patients had more than one chronic medical illness. Men and women were equally represented in the sample, and 45% had symptoms compatible with major depression. SES status based on residential income quintiles was evenly distributed across the subgroups. The mean MMSE score of subjects was 27/30 and the mean BNA score was 90/114. 22% of the sample was cognitively normal, 39% were demented (Alzheimer's disease, mixed dementia, dementia with lewy bodies, frontal-temporal dementia), 30% had mild cognitive impairment (MCI) and 9% had dementia secondary to some other cause (traumatic brain injury, lyme disease, cancer).

**Table 1:** Sample characteristics (N = 85)

Characteristics	N	Mean (SD)	Range
Age, in years	85	69.2(10.0)	50-89
Education, in years	84	14.0(3.5)	5-26
Cumulative Illness Rating	85	5.2(2.9)	0-12
Gender			
Male	48	50%	
Female	48	50%	
Depression			
Yes	38	44.70%	
No	47	55.30%	
Residential area SES			
Missing	4	4.70%	
Level 1 (low)	16	18.80%	
Level 2	11	12.90%	
Level 3	13	15.30%	
Level 4	11	12.90%	
Level 5 (high)	30	35.30%	
Diagnosis			
Normal	18	21.18%	
Alzheimer's Dementia	33	38.82%	
Mild Cognitive Impairment	26	30.59%	
Traumatic Brain Injury	2	2.35%	
Other	6	7.06%	
Behvioural Neurology Assessment	85	89.7(14.4)	59-111
Mini Mental State Examination Score	85	27(3)	16-30

In terms of comparative statistics, medical co-morbidity as measured by the CIRS score correlated negatively with the MMSE score (p < .001), BNA (p < .05) and all BNA sub scores (see Table 2). Conversely there was no correlation between medical co-morbidity and subjective memory complaints as measured by the PAOF (p > .05). Furthermore, there was no correlation with any of the PAOF sub scores (p > .05). Education correlated, as predicted, with cognition as measured by the MMSE (p < .05), but surprisingly not with the BNA or any of the sub scores. Residential SES correlated negatively with overall subjective memory complaints (p < .001), and with two sub scores, (memory complaints (p < .001) and language complaints (p < .01)) while it correlated positively with executive function (p < .01). Depression did not correlate significantly with any measure of objective cognitive function (p > .05) but positively with overall subjective general complaints (p < .001), memory complaints (p < .001) and language complaints (p < .05) with the exception of complaints in motor function (p > .05). Given that age may be a covariate or suppressor variable in the analyses, we reran the spearman rank correlations analyses with age as a co-variate and the results were similar except for a small change in the magnitude of the observed associations. In addition, we conducted multivariable linear regression to examine whether depression or subjective memory complaints are associated with objective memory/cognitive performance after the effects of medical co-morbidity are removed. We found no significant associations. We felt that it was important to run these additional analyses to ensure that the observed association between medical co-morbidity and objective memory impairment was not being driven by other confounders such as age, depression or subjective memory loss.

**Table 2:** Bivariate correlations between outcome variables

	Education years	Residential area SES	Cumulative illness rating	Depression
Subjective general complaints	-0.06	-0.37(***)	0.08	0.45(***)
Memory complaints	-0.05	-0.41(***)	0.07	0.45(***)
Language/communication problem	-0.08	-0.34(**)	0.03	0.25(*)
Complaints of motor difficulties	-0.03	-0.09	0.07	0.12
Complaints of thinking	0.06	-0.18	0.15	0.42(***)
Mini-Mental state examination	0.28*	0.04	-0.31(***)	-0.03
Behavioural Neurology Assessment (BNA) Total score	0.20	0.09	-0.26(*)	0.03
BNA Attention	0.10	0.08	-0.22(*)	0.14
BNA Memory	0.14	0.01	-0.28(*)	0.02
BNA verbal ability	0.20	0.07	-0.27(*)	0.02
BNA visuospatial function	0.15	0.05	-0.21(*)	-0.03
BNA brain's executive function	0.18	0.29(**)	-0.29(**)	0.05

Table 2 presents the Spearman correlations between the independent variables (e.g., level of residential area SES) and outcome variables (e.g., subjective complains and objective measures of cognitive abilities). Note: * p < .05, ** p < .01, *** p < .001.

## Discussion

We had predicted based on prior research that changes in objective cognitive function in our Memory Disorders Clinic sample would correlate strongly with increased medical co-morbidity, depression, low SES and low education. Surprisingly, decreased objective memory function correlated strongly with only medical co-morbidity, partially with education and not at all with residential SES. Subjective memory complaints did correlate with depression but not at all with medical co-morbidity and inversely with residential SES.

Prior research has suggested that SES correlates strongly with cognitive functioning [8]. It is possible in our study, given that we used residential SES as opposed to income levels as our measure of SES, that our sample size was not sufficiently large to detect differences. The weak link with education, however, is even more surprising given that low levels of education have been shown to be significantly associated with poor cognitive functioning. Education did correlate mildly with impaired objective memory performance as measured by the MMSE but did not correlate with a more specific measure of cognitive function, the BNA. Given that the sample looked at is quite elderly, it is possible that years of education is not a good proxy for level of intellectual function. The inverse relationship between subjective memory complaints and SES does suggest that patients with low SES tend to complain more about their memory function, the opposite trend from what would be observed using the theory of cognitive reserve.

Our study suggested a strong correlation between medical co-morbidity and objective memory function but a poor correlation with subjective memory function in our Memory Clinic sample. Thus, patients evaluated in our clinic with significant medical issues are clearly at risk for cognitive impairment. Subjective memory complaints are a measure of awareness of brain dysfunction. Awareness of such brain dysfunction is important as it may lead to the adoption of compensatory strategies, including use of calendars, aids, etc, which may enhance brain function. It may also make patients more likely to seek help or perhaps go on medications (such as cognitive enhancers) that may preserve their brain function. Previous studies have examined subjective memory complaints and have found a relatively poor correlation between complaints and actual cognitive performance [16,17]. This study contributes further to our understanding of the link between subjective memory complaints and objective memory function by demonstrating that this lack of association persists even in the context of significant medical illness.

It is interesting to speculate as to why subjective memory complaints would be low in patients with elevated medical co-morbidity, especially given the strong correlation with reduced cognitive performance. One potential explanation is that such patients may be so preoccupied with issues regarding their physical health that they have little awareness of cognitive issues. In addition, it is possible that it may have something to do with the theory of cognitive reserve [26]. According to this theory, it is possible that patients with high medical co-morbidity have low cognitive reserve to begin with and therefore experience cognitive changes in a more gradual way, resulting in poor awareness. Finally, it is possible that the relationship between medical co-morbidity and subjective memory complaints may be mediated by some other correlate, such as low SES or limited education. Our study in fact showed only a partial correlation with education and a negative correlation with SES, suggesting this is not likely the mechanism.

Interestingly approximately half of the patients in the clinic studied had a diagnosis of dementia while half did not. There is some evidence to suggest that patients with more severe cognitive impairment may be more inclined to underreport their cognitive impairment while the opposite is true of patients with less severe cognitive impairment. Thus, it is possible that diagnosis alone may explain to some degree the observed associations. Whatever the mechanism, the implications of these research findings are apparent. Patients evaluated in our Memory Disorders Clinic who are in a sense more impaired medically are less likely to be aware of their cognitive deficits, making them more vulnerable. Such patients may be less likely to seek help or develop compensatory strategies, resulting in elevated risk. At a clinical level, it may mean that such patient with high levels of medical co-morbidity need to be assessed differently, perhaps screened more rigorously for the presence of cognitive deficits, or the physician may need to make a greater effort to contact collateral sources. Medication compliance is an area where physicians need to be especially vigilant, as poor cognition may lead to poor compliance, resulting in a greater burden of medical illness.

This study has a number of limitations which should be discussed. First, all of the measurement scales used have some limitations. Specifically, the PAOF has not been validated in the elderly and the BNA is a compilation of tests that has not been correlated with other neuropsychological tests. However, the PAOF has been used in other cognitively impaired populations and the BNA has shown to be superior to the MMSE in detecting dementia. The CIRS is a good attempt to quantify medical co-morbidity but as with many scales may not capture the full impact of medical illness. Also, in our sample we looked at all patients and did not stratify based on diagnosis (cognitively normal, MCI, dementia) as we were more interested in looking at the effects of medical co-morbidity on overall subjective/objective memory function as opposed to diagnosis. Furthermore, the design of the study was cross sectional as opposed to longitudinal, the sample size was relatively small and patients were recruited from a Memory Disorders clinic as opposed to the community. Thus, the findings are not generalizable to other clinical settings such as the community, family practice setting, etc. Finally, while more sophisticated statistical methodology could be used to analyze the relationship between mood, medical co-morbidity and cognition we feel such analyses goes beyond the current scope of our paper given our sample size.

## Conclusions

In spite of these limitations, this study raises a number of issues that warrant further exploration. While it is clear from our preliminary findings that medical co-morbidity leads to low complaints and increased cognitive dysfunction, the precise mechanism of this is less clear. In addition, there are a number of health policy implications of our findings. Patients with high levels of medical co-morbidity should be screened more carefully for the presence of cognitive impairment and offered resources to help cope with this. Future studies should examine what the potential causes for our findings are and also look at how these research findings can be applied.

## Competing interests

The authors declare that they have no competing interests.

## Authors' contributions

CF was responsible for writing the article and assisting in interpretation of the data analyses. DJ was responsible for analyzing the data, defining the statistical approach and writing the statistical section of the manuscript. TS was responsible for critiquing the article, assisting with interpretation of the data analyses and aiding with the scientific design of the study. All of the authors have read and approved the final manuscript.

## Authors' information

CF is an adjunct scientist in the Li Ka Shing Knowledge Institute at St. Michael's Hospital in Toronto with an academic appointment of assistant professor at the University of Toronto Department of Psychiatry. DJ is a senior scientist and statistician at the University of Manitoba. TS is a scientist at the Li Ka Shing Knowledge Institute, St. Michael's Hospital and an assistant professor in the Department of Neurosurgery, University of Toronto.

## Pre-publication history

The pre-publication history for this paper can be accessed here:

http://www.biomedcentral.com/1471-2318/10/89/prepub
